# Epithelioid Hemangioma of the External Auditory Canal: A Case Report

**DOI:** 10.7759/cureus.115593

**Published:** 2026-09-01

**Authors:** Victoria Moens, Delphine Hoton, Frank Hammer, Laurence Boon, Sandra Schmitz

**Affiliations:** 1 Department of Ear, Nose and Throat, Cliniques Universitaires Saint-Luc, Brussels, BEL; 2 Department of Pathology, Cliniques Universitaires Saint-Luc, Brussels, BEL; 3 Department of Radiology, Cliniques Universitaires Saint-Luc, Brussels, BEL; 4 Division of Plastic Surgery, Université Catholique de Louvain, Brussels, BEL; 5 Department of Otolaryngology and Head and Neck Surgery, Cliniques Universitaires Saint-Luc, Brussels, BEL

**Keywords:** auricle angiomas, benign vascular lesion, epithelioid hemangioma, external ear, external ear mass, head and neck tumors

## Abstract

Epithelioid hemangioma (EH) represents a rare, benign neoplastic vascular proliferation that predominantly manifests within the cutaneous and subcutaneous tissues of the head and neck region. While well-documented in areas such as the scalp and forehead, its primary occurrence within the otolaryngologic tract, specifically involving the auricle and the cartilaginous portion of the external auditory canal, remains exceedingly uncommon. Due to its overlapping clinical and radiological features with other vascular and lymphoid pathologies, EH poses a significant diagnostic challenge for clinicians and pathologists alike. This case report highlights the importance of differential diagnosis with similar vascular and lymphoid conditions.

## Introduction

Epithelioid hemangioma is an infrequent, benign vascular tumor characterized by an abnormal proliferation of blood vessels. It is frequently located on and around the auricle. Clinically, lesions can appear at any age, grow slowly, and can be pruritic [1]. Its etiology remains idiopathic, although inflammatory responses, autoimmune factors, infectious agents, and potential genetic contributions cannot be entirely ruled out [2]. A definitive diagnosis is typically confirmed through histopathological examination via biopsy.

Given its rare presentation, its frequent location around the ear, and the potential for misdiagnosis as a malignant tumor, such as angiosarcoma, it is essential for ENT specialists to perform a comprehensive differential diagnosis to ensure precise and effective patient management. Through this case report, we aim to underscore the importance of differential diagnosis in evaluating vascular lesions in the head and neck region.

## Case presentation

We present the case of a 35-year-old woman referred to our ENT department due to progressively enlarging nodular lesions in the right ear canal that had continued to grow, leading to complete stenosis over a period of more than three years. The patient suffered from persistent itching, occasional sharp pain, and malodorous discharge, but no associated systemic symptoms. Clinical examination revealed the presence of multiple pinkish-red nodules (1 cm-2 cm) affecting the external auditory canal, the concha, the antihelix, and the antitragus, along with subcentimeter lymph nodes in the ipsilateral parotid and submandibular regions. The lesion was not warm, and there was no thrill on palpation.

The complexity of the case warranted a review at our multidisciplinary meeting, and further investigations were requested to establish a more precise diagnosis.

Imaging findings

Doppler ultrasound revealed a lesion with arterial and venous components, characterized by rapid arterial flow and systolic-diastolic modulations. Selective arteriography was subsequently performed. This showed a highly vascularized tumor supplied exclusively by a hypertrophied right posterior auricular artery. A dense arteriocapillary network with early venous drainage and a nodular appearance was noted. Although the vascular pattern raised the possibility of an arteriovenous malformation (AVM), the solitary arterial supply coupled with the nodular architecture was more suggestive of a hypervascular tumor rather than a true AVM (Figure 1).

**Figure 1:**
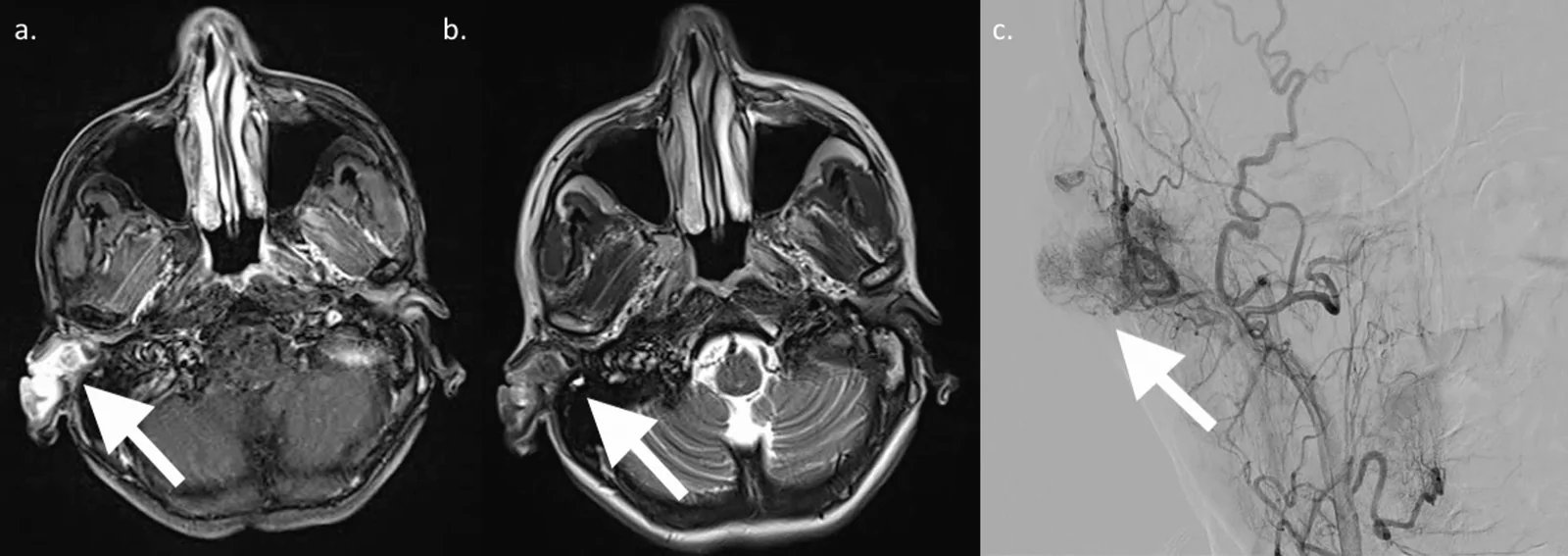
Imaging findings of an EH (see arrows) The MRI appearance of an EH as bright (white) on Gd T1 Dixon opp (a) and gray on T2 Dixon in (b) is a classic radiological finding. The bright signal on T1 reflects the intense contrast enhancement (Gd) due to the tumor’s high vascularity. Meanwhile, the gray signal on T2 indicates a solid, cellular tumor rather than a simple fluid-filled cyst or a common cavernous hemangioma. This combination confirms a highly vascularized, solid soft-tissue mass. On an arteriogram, an EH typically presents as a hypervascular mass with an intense and prolonged tumoral blush, enlarged feeding arteries, and dense capillary staining, which outlines the solid, cellular nature of the mass (c). EH, epithelioid hemangioma

MRI was conducted to assess the extent of the lesion. It confirmed the invasion of the right external auditory canal, concha, and antihelix, with a moderately hypointense signal on both T1- and T2-weighted sequences, along with marked contrast enhancement. The lesion measured 28 mm × 16 mm × 29 mm (transverse×anteroposterior×craniocaudal). Importantly, no abnormalities were detected in the regional lymph nodes; the parotid area and levels IIa and IIb all presented with subcentimeter lymph nodes (Figure 1).

Surgical management and histopathology

Following an initial shave biopsy, the lesion was surgically excised via resection of the auricle and the cartilaginous portion of the external auditory canal, achieving clear margins. The surgical specimen was subsequently submitted for histopathological analysis.

Microscopic examination revealed a lobular proliferation of well-formed small blood vessels lined by plump, epithelioid endothelial cells containing abundant eosinophilic cytoplasm and round, vesicular nuclei. These vessels were embedded in a loose stromal background and surrounded by a marked inflammatory infiltrate composed of lymphocytes, plasma cells, and numerous eosinophils. Prominent lymphoid follicles were also present (Figure 2). No significant cytological atypia or mitotic activity was identified. The underlying cartilage remained intact. The final histopathological report concluded with a diagnosis of EH.

**Figure 2:**
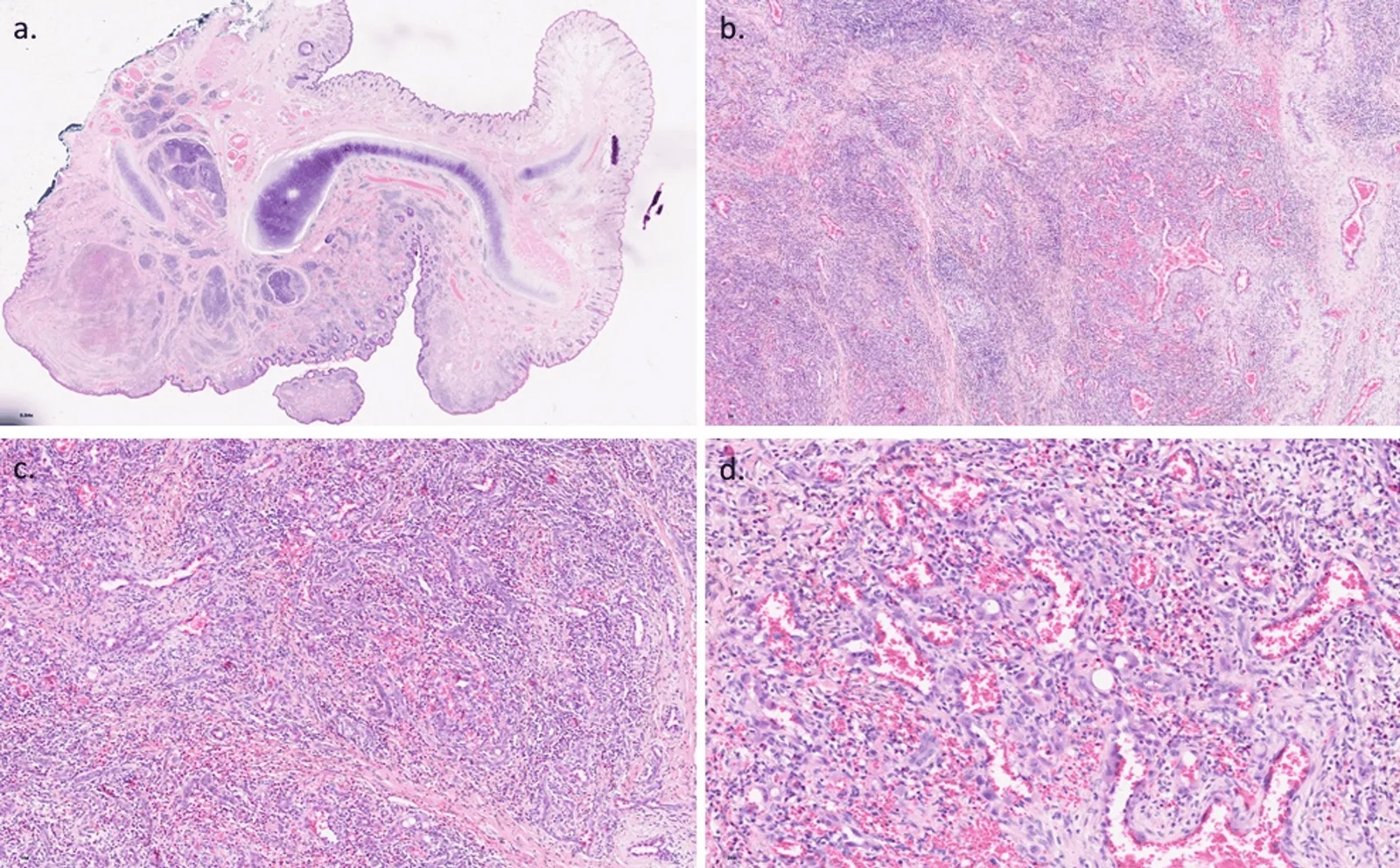
Histopathologic panoramic view of EH (a) H&E-stained panoramic view. (b) H&E stain, ×5 magnification. (c) H&E stain, ×10 magnification. (d) H&E stain, ×20 magnification. EH: epithelioid hemangioma; H&E: hematoxylin and eosin

Outcome

The patient was fitted with a custom-made ear epithesis, which provided excellent cosmetic results (Figure 3). At the three-year follow-up, there were no signs of local recurrence or systemic involvement.

**Figure 3:**
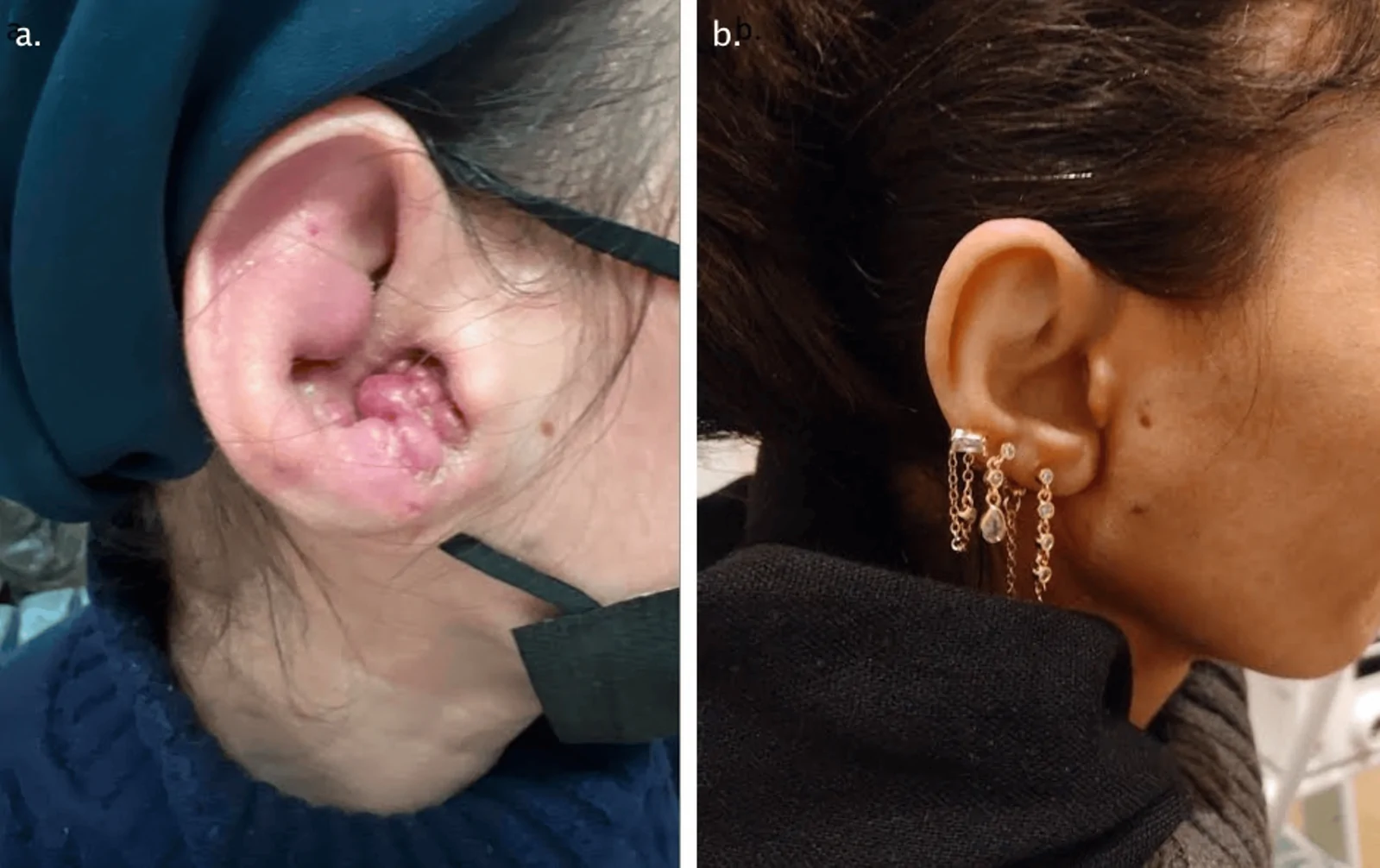
From preoperative lesion to postoperative prosthetic rehabilitation Preoperative state showing extensive EH involving the entire auricle and ear canal, with purplish nodules (a). Postoperative outcome following reconstruction with an auricular epithesis (b). EH, epithelioid hemangioma

## Discussion

EH, historically referred to as angiolymphoid hyperplasia with eosinophilia (ALHE), is an uncommon vascular lesion first described in 1969 by Wells and Whimster [1]. Initially thought to be a late-stage manifestation of Kimura’s disease, EH is now widely accepted as a distinct clinical and histopathological entity [1,2]. 

It primarily affects the skin and subcutaneous tissues of the head and neck region, especially around the ears, scalp, and face [3]. Clinically, EH manifests as one or more firm, erythematous to violaceous papules or nodules, typically ranging from a few millimeters to several centimeters in diameter [4]. These lesions are often associated with pruritus, occasional ulceration, and, in rare cases, localized pain, which may arise from secondary infection, as observed in our case. Additionally, regional infracentimetric lymphadenopathy may be noted [5].

EH primarily affects individuals in their third to fourth decade of life, with no significant sex predilection. It appears to be more prevalent among Asian populations, followed by Caucasians, and is less frequently observed in individuals of African descent [3].

The exact pathogenesis of EH remains incompletely understood, and its biological nature has long been debated, with earlier studies proposing either a benign vascular neoplasm or a reactive inflammatory vascular proliferation. Increasing molecular evidence, however, strongly supports a neoplastic origin. Recurrent FOS and FOSB gene rearrangements have been identified in a substantial proportion of EHs, particularly those arising in bone and deep soft tissues, demonstrating the clonal nature of the lesion and providing compelling evidence for its classification as a benign vascular neoplasm rather than a purely reactive process [6,7]. Earlier observations also reported an association with arteriovenous shunts in approximately 43% of cases, suggesting that local hemodynamic changes may contribute to tumor development. Hyperestrogenic conditions, which promote angiogenesis similarly to pyogenic granuloma, have also been proposed as potential contributing factors [8,9]. In addition, renin expression within associated arteriovenous shunts has been hypothesized to stimulate eosinophilic recruitment and angiogenesis, although this mechanism remains to be fully elucidated [2,8].

Conversely, a reactive inflammatory component is supported by reports of EH developing after local trauma, including surgical procedures, frostbite, and lacerations, suggesting that tissue injury may act as a triggering event in susceptible individuals [10]. Nevertheless, the identification of recurrent FOS/FOSB rearrangements has shifted the current consensus toward considering EH a benign clonal vascular neoplasm with secondary inflammatory features rather than a primary inflammatory disorder [6-8,11].

The diagnosis of EH is established by histopathological examination. Microscopically, the lesion is characterized by well-formed capillary-sized vessels lined by plump epithelioid endothelial cells with abundant eosinophilic cytoplasm and vesicular nuclei. These vascular channels are embedded within a loose fibromyxoid stroma containing a prominent inflammatory infiltrate composed predominantly of lymphocytes, eosinophils, and plasma cells. Cytological atypia, significant mitotic activity, and necrosis are typically absent, supporting the benign nature of the lesion [12,13].

Immunohistochemically, EH demonstrates strong and diffuse expression of endothelial markers, including cluster of differentiation (CD)31, ERG, and FLI1, which are considered the most sensitive markers for confirming endothelial differentiation. Factor VIII-related antigen (von Willebrand factor) is usually positive but is less sensitive than CD31. In contrast, CD34 expression is variable and is often weak, focal, or absent in epithelioid endothelial cells, making it less reliable as a diagnostic marker. More recently, diffuse nuclear expression of FOS and/or FOSB has emerged as a highly valuable diagnostic marker, reflecting the underlying recurrent gene rearrangements and helping distinguish EH from other epithelioid vascular neoplasms, particularly epithelioid hemangioendothelioma and epithelioid angiosarcoma (Table 1) [8,10-14].

**Table 1:** Recommended immunohistochemical and molecular markers for the diagnosis of EH FLI1, Friend leukemia integration 1 transcription factor; FISH, fluorescence in situ hybridization; EH, epithelioid hemangioma; CD, cluster of differentiation

Diagnostic feature	Expression in EH	Diagnostic significance	Key references
CD31	Strong and diffuse membranous staining	The most sensitive endothelial marker; confirms vascular differentiation	[10,12]
ERG	Strong and diffuse nuclear staining	A highly sensitive and specific endothelial transcription factor; routinely recommended to confirm endothelial lineage	[12]
FLI1	Diffuse nuclear positivity	A sensitive endothelial marker, complementary to ERG and CD31	[12,13]
CD34	Variable; often weak, focal, or absent in epithelioid endothelial cells	A supportive endothelial marker, but less consistently expressed than CD31 or ERG	[10,12,13]
Factor VIII (von Willebrand factor)	Positive	A classic endothelial marker; less sensitive than CD31 but supports vascular differentiation	[2,10,12,13]
FOS and/or FOSB	Strong nuclear immunoreactivity	A highly characteristic finding reflecting recurrent FOS/FOSB gene rearrangements; particularly useful in challenging cases	[6-9]
FOS/FOSB gene rearrangements	Recurrent clonal rearrangements detected by FISH or RNA sequencing	A molecular hallmark of many EHs; highly useful for diagnosis, particularly in bone and soft-tissue lesions	[8,12,14]

A critical differential diagnosis is Kimura’s disease, which typically affects young Asian males and presents as deeper subcutaneous masses, often involving the salivary glands and accompanied by systemic manifestations such as peripheral eosinophilia, elevated IgE, and nephrotic syndrome. Histologically, Kimura’s disease shows prominent lymphoid follicles and lacks the vascular proliferation seen in EH [15].

The prognosis for EH is excellent, given that it is a benign condition. While instances of spontaneous regression have been documented, such occurrences remain rare.

Currently, there are no standardized treatment guidelines for EH. In the largest systematic review of ALHE/EH, which included 908 patients and predominantly involved head and neck lesions, complete surgical excision was identified as the treatment of choice, providing the longest disease-free survival (mean, 4.2 years) and the lowest recurrence rate among all therapeutic modalities [3]. Recurrence was more frequently associated with younger age, longstanding disease, multiple or bilateral lesions, and incomplete excision rather than with aggressive biological behavior. More recently, a multicenter retrospective study reported an overall local recurrence rate of 18.6% after long-term follow-up, confirming that recurrence is uncommon but not exceptional [16].

Alternative therapies for patients with cosmetically sensitive lesions or recurrence include laser therapies, although these have recurrence rates exceeding 50%. Other reported modalities include photodynamic therapy, cryotherapy, radiotherapy, electrodesiccation, sclerotherapy (e.g., with bleomycin or polidocanol), and radiofrequency ablation, but data remain limited [3]. Topical treatments with corticosteroids, tacrolimus, or timolol have shown limited long-term efficacy. Systemic therapies such as isotretinoin, corticosteroids, and pentoxifylline have generally been ineffective [10]. Although several case reports indicate that beta-blockers (propranolol) may reduce or completely resolve lesions, large-scale randomized controlled trials are still needed to confirm these findings [17].

## Conclusions

EH is a rare, benign vascular neoplasm that frequently involves the head and neck region and may present a diagnostic challenge because of its variable clinical appearance and overlap with vascular malformations and other benign or malignant auricular lesions. Definitive diagnosis relies on histopathological examination, supported by immunohistochemical and, when necessary, molecular findings, while imaging is valuable for assessing lesion extent and vascular characteristics. Complete surgical excision with negative margins remains the treatment of choice and offers excellent long-term outcomes despite the potential for local recurrence.

This case highlights the importance of including EH in the differential diagnosis of auricular vascular lesions and underscores the advantages of a multidisciplinary approach involving discussions among physicians specializing in vascular anomalies to optimize the management of such cases.
